# Probing the chemical constituents of *Cassia javanica* and its *in vitro* analyses as a potent drug

**DOI:** 10.1098/rsos.211626

**Published:** 2022-01-26

**Authors:** Muhammad Abdul Haleem, Qurrat ul-Ain, Muhammad Saadiq, Muhammad Iqbal, Hussain Gulab, Sajid Ali, Sabir Khan

**Affiliations:** ^1^ Department of Chemistry, Bacha Khan University, Charsadda 24420, KPK, Pakistan; ^2^ Laboratory of Physical Chemistry Research, Faculty of Sciences, National University of Engineering, Av. Tupac Amaru 210, Lima 25, Peru

**Keywords:** *Cassia javanica*, *Proteus mirabilis*, *Klebsiella pneumoniae*, dpph and MIC, MBC

## Abstract

The phytochemical screening of the crude methanolic stem extracts of *Cassia javanica* plant showed the presence of different classes of organic compounds like alkaloids, tannins, flavonoids, saponins, phlobatanins, steroids, anthraquinone and cardiac glycoside. The starching frequencies of these functional groups were determined from FT-IR spectroscopic data. The crude and their fractions were examined for antibacterial potential against *Klebsiella pneumoniae* and *Proteus mirabilis*. The antibacterial assay showed maximum zone of inhibition for ethyl acetate fraction, i.e. 20 mm against *Proteus mirabilis* and 18 mm against *Klebsiella pneumoniae* in the comparison with Levofloxacin used as standard (40 mm). Meanwhile for methanolic crude extract, the inhibition zone was recorded 14 mm against *Klebsiella pneumoniae* and 22 mm against *Proteus mirabilis*. The minimum inhibitory concentrations and minimum bactericidal concentrations were recorded as 187.5 µg ml^−1^ against *Proteus mirabilis* and 93.75 µg ml^−1^ against *Klebsiella pneumoniae.* The scavenging free radical assay was noted as 69.61% at the concentration of 100 ppm.

## 1. Introduction

Plants sustain life on Earth, as they provide all the basic needs of life like food, shelter and medicines etc. [1,2]. Plants which are used in order to cure or prevent a disease or to alter pathological and physiological process, or any plant working as a source of drugs or their precursors are known as medicinal plants [3,4]. Most herbal products are present in the form of leaf powder, pills and paste for the treatment of several diseases in humans [5]. These medicinal plants contain active metabolites in different parts like roots, stem, bark and leaves [6]. The roots of the *Combretum caffrum* African Bush Willow were commonly used in traditional pain medicine [7]. It was estimated that over 50% of the available drugs were derived from medicinal plants [8]. Salicylic acid, a painkiller, is a precursor of aspirin which was originally isolated from white wallow bark [9]. Cinchona bark is the source of quinine, an antimalarial drug [10]. Morphine, another important secondary metabolite, belongs to the alkaloid class and was isolated from opium poppy which has been used for its analgesic potential and for many other diseases. Phytochemical investigation of *Carica Papaya* leaf extract exhibited the existence of glycosides, flavanoids, alkaloids, saponins, tannins, phenols and steroids etc. Medicinally, *C. Papaya* is used as an anti-cancer agent [11].

*Cassia fistula* flowers showed several pharmacological activities. The ethyl acetate fraction of flower showed antifungal activity due to the existence of Rhein [12]. Similarly, the methanolic extract of leaves, seed and fruit of *Carica papaya* Linn showed significant anti-inflammatory, antimicrobial and antioxidant activity [11]. Similarly, ethanolic seed extract of *Cassia tora* showed many biological activities including spasmogenic, anti-inflammatory, antifungal and hypolipidemic [13,14]. *Ageratum conyzoides* Linn plant leaves and flowers ethanolic extract showed significant antioxidant activity [15]. Similarly, *Fumaria capreolata* and *Fumaria bastardii* exhibited a strong antioxidant activity. These were used as natural and good sources of antioxidants [16].

*Cassia javanica* is a member of sub-family caesalpinioideae and family Leguminosae (Fabaceae) [14]. *C. javanica* is naturally distributed from India to Malaysia, Southern China and the Phillipines. It is also cultivated in tropical regions of Asia. *C. javanica* has several pharmacological properties such as antioxidant, antidiabetic, anti-cancer and antimicrobial activities [17]. The aim of the present study is to examine the qualitative phytochemical screening and antibacterial activity of the stem extracts of *C. javanica*.

## 2. Material and methods

### 2.1. Materials

Dichloromethane (DCM), *n-*hexane, ethyl acetate (commercial grade), ammonium hydroxide, ammonia, acetic anhydride, sodium hydroxide (NaOH), ferric chloride, Dragendroff''s reagent, H_2_O (distilled), H_2_SO_4_, HCl, glacial acetic acid, chloroform and DPPH (Sigma Aldrich) were used for phytochemical screening. Nutrient agar (Bio world) and nutrient broth (Merck) were used for biological activities.

### 2.2. Plant material

Based on its ethno-medicinal properties, *C. javanica* was collected from Shalman park Hayat Abad Peshawar, Khyber-Pakhtunkhwa, Pakistan in November, 2018, and identified by Dr Waheed, Botany Department of B.K.U.C. The specimen of *C. javanica* was also submitted to the herbarium of Bacha Khan University, Charsadda (voucher N0: HBKU-702).

### 2.3. Preparation of extracts

*Cassia javanica* plant stem was shad dried. The dried plant material (stem) of *C. javanica* was ground to make a fine powder. In methanol, these dried materials were soaked for one week and subjected to extraction. The extract was then concentrated under reduced pressure using a rotary evaporator (Stuart RE300DB) at 45°C. The extract was fractionated into polar (methanol, ethyl acetate and DCM) and non-polar (*n-*hexane) solvents by separating funnel.

### 2.4. Phytochemical screening

The phytochemical screening was performed for qualitative identification of phytochemical constituents in crude (methanolic), *n*-hexane fraction, ethyl acetate, and DCM fractions of *C. javanica* using standard protocol [18].

### 2.5. FTIR analysis

IR was performed on a Perkin Elmer instrument. The scanning range of IR frequencies was from 400 to 4000 cm^−1^.

### 2.6. Antibacterial assay

The antibacterial activity of the fraction of *C. javanica* was examined using agar well diffusion method against clinically isolated pathogenic bacteria e.g. *Klebsiella pneumoniae* and *Proteus mirabilis*.

One millilitre of the broth inoculum of the test bacteria was taken with the help of sterile micropipette and spread equally with the help of a sterile glass rod spreader, and the plates were allowed to dry at room temperature. Subsequently, wells of 6 mm diameter were bored in each plate. Sixty-six microlitres of compound (3 mg ml^−1^ of DMSO) was added to each well using a sterilized micropipette and allowed to diffuse for 2 h. The plates were incubated at 37°C for 24 h and the zone of inhibition was calculated in mm. Levofloxacin, Ciprofloxacin, Amoxicillin and Norfloxacin were used as standard antibiotics [19].

#### 2.6.1. Minimum inhibitory concentrations

To determine the minimum inhibitory concentration (MIC) and minimum bactericidal concentration (MBC) of different strains, freshly prepared tubes containing serial twofold dilutions in 2 ml of nutrient broth (range, 1000 µg ml^−1^ to 1.95 µg ml^−1^) was inoculated underneath the surface with 5 × 10^5^ to 1 × 10^6^ cells in 0.2 ml of nutrient broth mixed by flushing and incubated without shaking. After 24 h of incubation, all broths were examined for visual turbidity. The MIC was determined by visual examination for turbidity after 24 h of incubation at 37°C.

#### 2.6.2. Minimum bactericidal concentrations

An MBC was performed by using fresh agar plates to check whether fractions were either bacteriostatic or bactericidal. The MBC was considered the lowest concentration of the test plant fractions which prevented the growth and reduced the inoculums by greater than or equal to 99.9% within 24 h [20].

### 2.7. DPPH scavenging free radical assay

In order to evaluate the antioxidative ability of the plant against 2,2-diphenyl,1-picryl- hydrazine, methanolic solution of DPPH was allowed to react with different concentrations of the crude fractions of *C. javanica.* A 50 ppm solution of DPPH was prepared (in methanol). One millilitre of DPPH solution was added to 4 ml of sample solution (in methanol), comprising 10 to 100 ppm, and to the control solution. The sample was incubated for 30 min in the dark at 25°C and then the % inhibition was determined at 517 nm. Ascorbic acid was used as standard. Per cent radical scavenging activity (%RSA) was calculated as follows [21].

$$\text{\%}\mathrm{DPPH}=\frac{(\mathrm{OD}\;\mathrm{Control}\text{–}\mathrm{OD}\;\mathrm{sample})\times 100}{\mathrm{OD}\;\mathrm{Control}}.$$

## 3. Results and discussion

### 3.1. Phytochemical profiling

Phytochemical analysis of plant species *C. javanica* was screened for phytochemical components like alkaloids, flavonoids, phlobatanins, anthraquinone, saponins, tannins and steroids. Different fractions (polar and non-polar) of crude extract of *C. javanica* showed different chemical profile; e.g. in crude extract (methanolic) tannins, flavonoids, phlobatanins and anthraquinone were present while cardiac glycoside does not express its presence. *n*-hexane fraction contains tannins and steroids while alkaloids, flavonoids, steroids, cardiac glycoside, anthraquinone and phlobatanins were absent in *n-*hexane fraction. In DCM fraction, the tannins and steroids tests were positive, alkaloids, flavonoids, saponins, steroids, cardiac glycoside, anthraquinone and phlobatanins tests were negative. On the other hand, ethyl acetate fraction showed positive test for alkaloids, tannins, flavonoids, cardiac glycoside, anthraquinone and phlobatanins (table 1).

**Table 1.** Phytochemical screening results of the crude extract and different fractions of *C. javanica*. + indicate present and − indicate = absent.

phytochemical constituents	crude extract	ethyl acetate	DCM	n-hexane
alkaloids	−	+	−	−
tannins	+	+	+	+
flavonoids	+	+	−	−
saponins	−	−	−	−
phlobatanins	+	+	−	−
steroids	−	−	+	+
anthraquinone	+	+	−	−
cardiac glycoside	−	+	−	−

The yields of phytochemicals present in *C. javanica* indicate that a total of 9% of the dry mass of *C. javanica* is composed of active phytochemicals/metabolites. Similarly, the yield per dry mass of *C. javanica* indicates that most of the metabolites were drained to ethyl acetate fraction because the crude yield obtained from *C. javanica* was 90 g, out of which 55 g was only ethyl acetate fraction which is 61.10% of total crude obtained, while the yield for DCM fraction was 17.78% and 21.12% for *n*-hexane fraction (table 2). The high per dry mass yield percentage of ethyl acetate fraction also suggests that aforementioned metabolites possess either polar functionalities or high polar moieties. Therefore, most of the phytochemicals were mainly fractionated in a polar solvent instead of less polar (DCM) or non-polar solvent (*n*-hexane). The nature of these functionality/moieties was characterized using FT-IR analysis.

**Table 2.** Yield of crude/fraction per dry mass of *C. javanica*.

weight of plant powder	solvent	crude/fraction	yield in gram
1 Kg	methanol	crude	90
	ethyl acetate	fraction	55
	DCM	fraction	17
	*n*-hexane	fraction	21

### 3.2. FT-IR analysis

**Figure 1.**
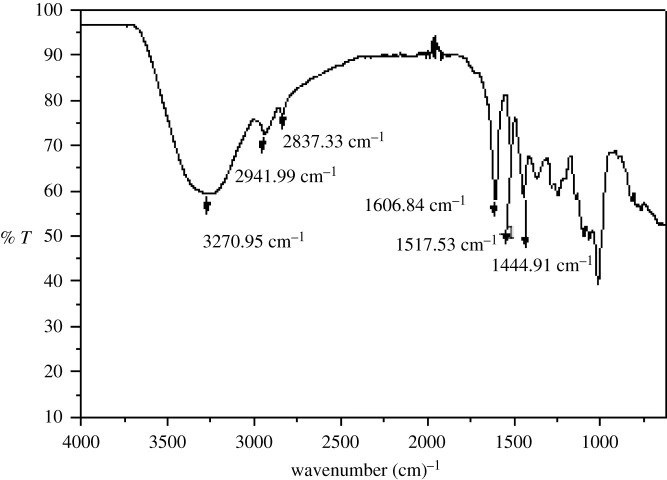
FT-IR spectrum of ethyl acetate fraction of *C. javanica* extracts showing absorption bands pertaining to different functional group.

The FT-IR spectrum (figure 1) of *C. javanica* exhibits a broadband at 3270 cm^−1^ indicating the presence of phenolic –OH group. At 1606 cm^−1^, a sharp band was indicated NH bending (aliphatic amides). The sharp band at 1517 cm^−1^ was indicated NH bending (primary amine). The characteristic absorption band of C-H stretching appeared at 2941 cm^−1^ and 2837 cm^−1^. The broadband ranges from 2500 cm^−1^ to 3500 cm^−1^ and was assigned H-bonded phenolic (-OH).

The above IR characterization indicates the presence of the alcoholic, carboxylic acids, amine and amidic functionalities. All these characterized functionality (-COOH, -OH, -NH and –CONH-) are polar in nature and hence mainly found in ethyl acetate fraction of the crude extract. These analyses (phytochemical, yield calculation and FT-IR data) indicate the presence of the above-mentioned bioactive compounds (table 1) because most of the metabolites possess these functional groups.

The development of resistant bacteria to the currently available antibiotics is an issue of great concern for public health. Nowadays the development of multi-drugs-resistant (MDR) bacteria is a major issue both in hospitals and in communities. Novel antibacterial medications are needed to cure the infections effectively caused by these MDR bacteria. In this regard, the medicinal plants have been extensively evaluated to determine their antibacterial efficacy which can finally be developed as a good antibacterial agent for the management of bacterial infections. A large number of plant crude extracts, isolated compounds and synthetic organic molecules have been extensively studied to be effective antimicrobial agents against MDR bacterial strains [22,23]. Keeping in view the significance of medicinal plants as an antimicrobial agent, the crude extract and fractions of *C. javanica* were screened for their antimicrobial potential against clinically isolated pathogenic bacteria.

#### 3.2.1. Antibacterial potential of crude (methanolic) extract

**Figure 2.**
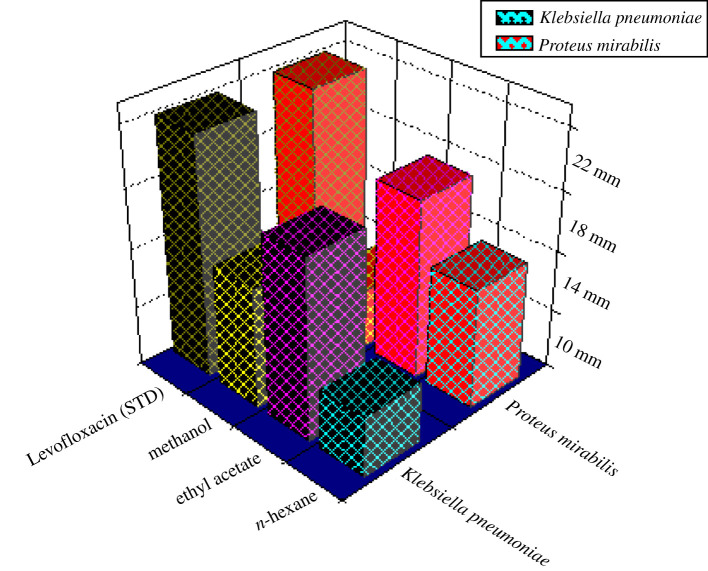
Antibacterial assay of methanolic, ethyl acetate and *n*-hexane fraction.

Methanolic crude extract showed 22 mm inhibition against *P. mirabilis*. Levofloxacin (standard) exhibited 40 mm inhibition against *P. mirabilis*. While methanolic crude fraction showed 14 mm inhibition against *K. pneumoniae*. Similarly, Levofloxacin exhibited 22 mm against *K. pneumoniae*. These results showed that the antibacterial activity of crude was not significant (figure 2). As mentioned in table 1, the alkaloids and cardiac glycoside does not express its presence in the crude extract; therefore, it may be suggested that the absence of these classes of organic compounds is mainly responsible for non-significant antibacterial activity.

#### 3.2.2. Antibacterial potential of ethyl acetate fraction

Twenty millimetres zone of inhibition of ethyl acetate fraction was recorded against *Proteus mirabilis*. Levofloxacin (standard) exhibited 40 mm against *P. mirabilis*. Similarly, 18 mm of inhibition was recorded against *K. pneumonia* for ethyl acetate fraction, while Levofloxacin (standard) exhibited 22 mm inhibition zone against *K. pneumoniae*. These results indicated that ethyl acetate fraction of *C. javanica* stem exhibited significant activity (figure 2).

#### 3.2.3. Antibacterial potential of *n-*hexane fraction

Zone of inhibition noted for *n*-hexane fraction was 12 mm against *P. mirabilis.* Levofloxacin (standard) exhibited 40 mm *P. mirabilis*. Similarly, *n-*hexane fraction showed 10 mm against *K. pneumoniae*. Similarly, Levofloxacin (standard) exhibited 22 mm inhibition against *K. pneumoniae*. *C. javanica* stem *n-*hexane fraction exhibited non-significant potential (figure 2). The non-significant activity may be associated with the absence of organic constituents like alkaloids, flavonoids, saponins, steroids, cardiac glycoside, anthraquinone and phlobatanins

#### 3.2.4. Antibacterial potential of dichloromethane fraction

The DCM fraction of *C. javanica* stem against pathogenic bacteria exhibited no zone of inhibition. Levofloxacin (standard) exhibited (40 mm) against *P. mirabilis*. Similarly, Levofloxacin (standard) (22 mm) zone of inhibition against *K. pneumoniae*. These results showed that DCM fraction of *C. javanica* also exhibits non-significant activity (figure 2). The non-significant activity may be associated with the absence of organic constituents like alkaloids, flavonoids, saponins, steroids, cardiac glycoside, anthraquinone and phlobatanins.

These results indicate that only ethyl acetate fraction has significant antibacterial activity (18 mm of zone inhibition as compared to standard) while all other fractions are not significant (figure 2). The significant antibacterial activity of ethyl acetate is due to the presence of alkaloid and cardiac glycoside because other phytochemical constituents like tannins, flavonoids, phlobatanins, steroids and anthraquinone are also present in a crude extract of the stem. The significance in the antibacterial activity of the ethyl acetate fraction may be associated with the presence of bioactive compounds like alkaloids, anthraquinone, flavonoids and cardiac glycoside. Due to these phytochemical constituents, the ethyl acetate fraction showed significant activity (figure 2). Therefore, MIC and MBC analyses was carried out only for ethyl acetate fraction.

### 3.3. Minimum inhibitory concentrations and minimal bacterial concentrations

MIC of ethyl acetate fraction of *C. javanica* along with positive and negative control against the selected pathogen has been listed in table 2. The range of MIC of the ethyl acetate fraction observed was 1000–1.95 µg ml^−1^. The MIC value exhibited by ethyl acetate fraction was 187.5 µg ml^−1^ against *P. mirabilis* and 97.75 µg ml^−1^ against *K. pneumoniae* as compared to Levofloxacin which suggests a good activity. Similarly, the MBC value was 94.5 µg ml^−1^ against *P. mirabilis* and 50.25 against *K. pneumonia* in comparison with Levofloxacin (table 3).

**Table 3.** MIC and MBC values of various fractions of *C. javanica*.

s. no	sample	*Proteus mirabilis*		*Klebsiella pneumoniae*	
		MIC (µg ml^−1^)	MBC (µg ml^−1^)	MIC (µg ml^−1^)	MBC (µg ml^−1^)
1	ethyl acetate	187.5	94.5	97.75	50.25
2	crude extract	—	—	—	—
3	DCM	—	—	—	—
4	*n*-hexane	—	—	—	—
5	levofloxacin	46.87	23.43	93.75	46.87

### 3.4. Antioxidant potential

The maximum % scavenging effect noticed for ethyl acetate fraction was 45.00, 48.9, 49.70, 52.56, 53.28, 57.02, 59.65, 62.2, 65.23and 69.61% at concentrations of 10 µg ml^−1^, 20 µg ml^−1^, 30 µg ml^−1^, 40 µg ml^−1^, 50 µg ml^−1^, 60 µg ml^−1^, 70 µg ml^−1^, 80 µg ml^−1^, 90 µg ml^−1^ and 100 µg ml^−1^. The antioxidant potential of crude extract was 20.5, 22.26, 27.08, 29.98, 30.46, 30.58, 31.30, 31.85, 35.04 and 36.00% at concentrations of 10 µg ml^−1^, 20 µg ml^−1^, 30 µg ml^−1^, 40 µg ml^−1^, 50 µg ml^−1^, 60 µg ml^−1^, 70 µg ml^−1^, 80 µg ml^−1^, 90 µg ml^−1^ and 100 µg ml^−1^. The antioxidant effect of *n*-hexane fraction was 4.77, 4.97, 5.41, 5.93, 6.45, 6.61, 6.69, 7.04, 7.84 and 8.84% at concentrations of 10 µg ml^−1^, 20 µg ml^−1^, 30 µg ml^−1^, 40 µg ml^−1^, 50 µg ml^−1^, 60 µg ml^−1^, 70 µg ml^−1^, 80 µg ml^−1^, 90 µg ml^−1^ and 100 µg ml^−1^. The antioxidant effect of DCM fraction was 2.07, 3.82, 4.06, 4.38, 4.46, 4.61, 4.73, 4.77, 5.13 and 5.65% at tested concentrations of 10, 20, 30, 40, 50, 60, 70, 80, 90 and 100 µg ml^−1^. While scavenging free radical effect of ascorbic acid (standard) was 77.5, 80.77, 81.3, 82.1, 84.57, 84.85, 87.42, 87.75, 88.9 and 90.65 at tested concentrations of 10, 20, 30, 40, 50, 60, 70, 80, 90 and 100 ppm.

**Figure 3.**
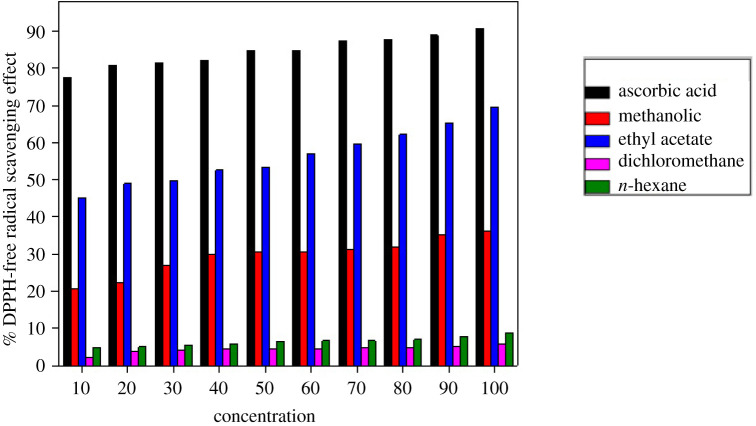
DPPH scavenging free radical potential of different fractions of *C. javanica*.

From the above results, it can be concluded that ethyl acetate showed better antioxidant activity, i.e. 69.61% at concentration of 100 ppm as compared to other fractions like *n*-hexane, which showed 8.84% at concentration 100 ppm. DCM showed 5.65% at concentration 100 ppm and crude extract showed activity of 36.00% relative to standard ascorbic acid (90.65 at concentration 100 ppm). Thus, DPPH scavenging effect of *n*-hexane and DCM fractions exhibited lesser activity as compared to methanolic crude extract and ethyl acetate fraction (table 4 and figure 3).

**Table 4.** Free radical scavenging activity of crude and different fractions of *C. javanica.*

concentration of sample (ppm)	ascorbic acid (STD)	crude (methanolic)	Et.Acetate fraction	DCM fraction	*n-*hexane fraction
10	77.5	20.5	45.00	2.07	4.77
20	80.77	22.26	48.9	3.82	4.97
30	81.3	27.08	49.70	4.06	5.41
40	82.1	29.98	52.56	4.38	5.93
50	84.57	30.46	53.28	4.46	6.45
60	84.85	30.58	57.02	4.61	6.61
70	87.42	31.30	59.65	4.73	6.69
80	87.75	31.85	62.2	4.77	7.04
90	88.9	35.04	65.23	5.13	7.84
100	90.65	36.00	69.61	5.65	8.84

The emergence of the drug-resistant strains made the situation more alarming. It has been proved that the development of biofilm by the bacteria makes them more resistant to the available antibiotics both *in vitro* and *in vivo* [24]. As mentioned earlier, the main objectives of the current work were to evaluate the photochemistry of *C. javanica* and to find the antibacterial potential of crude and fractions against clinically isolated pathogenic bacteria. Our results showed that only ethyl acetate fraction of *C. javanica* exhibited significant antibacterial potential against *P. mirabilis* and *K. pneumonia* (18 mm of zone of inhibition) as compared to other fractions (figure 2).

MIC is the lowest concentration of an antibacterial agent that the visible growth of microorganism after overnight incubation will inhibit and (MBC) as the lowest concentration of antibacterial agent that will prevent the growth of an organism subculture onto antibiotic-free media. The MIC and MBC value of the tested fraction was significant and was comparable with standard antibiotics (table 3). Similarly, ethyl acetate fraction also showed better antioxidant activity (69.61%) at a concentration (100 ppm) as compared to other fractions (table 4).

## 4. Conclusion

The phytochemical screening of *C. javanica* plant stem extract showed the presence of chemical constituents, i.e. alkaloids, tannins, flavonoids, saponins, phlobatanins, steroids, anthraquinone and cardiac glycoside that need to be isolated and pass through a clinical trial in order to explore its chemical structure for mapping medicinal chemistry. Moreover, ethyl acetate fraction is comprised of alkaloids and cardiac glycoside. Due to the presence of phytochemical constituents, like alkaloids and cardiac glycoside, it exhibited significant antibacterial activity with reasonable MIC and MBC values. Moreover, the antioxidant activity of the same fraction was also significant as compared with other fractions. This study shows that the test ethyl acetate fraction can be used as a potent antibacterial agent in implant-associated infections; however, further *in vivo* studies need to be employed to be used as an anti-infective agent for the management of pathogenesis caused by bacteria.

From our results, it can be concluded that ethyl acetate extract of *C. javanica* comprises certain types of alkaloids and cardiac glycoside that will be the new competitor for existing antibiotic drugs.

## Data accessibility

This article has no additional data.
